# QP-Adaptive Dual-Path Residual Integrated Frequency Transformer for Data-Driven In-Loop Filter in VVC

**DOI:** 10.3390/s25134234

**Published:** 2025-07-07

**Authors:** Cheng-Hsuan Yeh, Chi-Ting Ni, Kuan-Yu Huang, Zheng-Wei Wu, Cheng-Pin Peng, Pei-Yin Chen

**Affiliations:** Department of Computer Science and Information Engineering, National Cheng Kung University, Tainan 70101, Taiwan; p78121530@gs.ncku.edu.tw (C.-H.Y.); dfdf22222@gmail.com (C.-T.N.); kyhuang@nkust.edu.tw (K.-Y.H.); tankwu@gmail.com (Z.-W.W.); kus0829@gmail.com (C.-P.P.)

**Keywords:** CNN, H.266/VVC, in-loop filter, IoT video coding, embedded AI

## Abstract

As AI-enabled embedded systems such as smart TVs and edge devices demand efficient video processing, Versatile Video Coding (VVC/H.266) becomes essential for bandwidth-constrained Multimedia Internet of Things (M-IoT) applications. However, its block-based coding often introduces compression artifacts. While CNN-based methods effectively reduce these artifacts, maintaining robust performance across varying quantization parameters (QPs) remains challenging. Recent QP-adaptive designs like QA-Filter show promise but are still limited. This paper proposes DRIFT, a QP-adaptive in-loop filtering network for VVC. DRIFT combines a lightweight frequency fusion CNN (LFFCNN) for local enhancement and a Swin Transformer-based global skip connection for capturing long-range dependencies. LFFCNN leverages octave convolution and introduces a novel residual block (FFRB) that integrates multiscale extraction, QP adaptivity, frequency fusion, and spatial-channel attention. A QP estimator (QPE) is further introduced to mitigate double enhancement in inter-coded frames. Experimental results demonstrate that DRIFT achieves BD rate reductions of 6.56% (intra) and 4.83% (inter), with an up to 10.90% gain on the BasketballDrill sequence. Additionally, LFFCNN reduces the model size by 32% while slightly improving the coding performance over QA-Filter.

## 1. Introduction

Emerging technologies, including the Internet of Things (IoT), artificial intelligence (AI), and others, are gradually contributing to improvements in human lifestyles. The widespread deployment of IoT devices has led to their close integration with various aspects of daily life, allowing not only human–device interaction but also inter-device communication for more collaborative services [1,2]. With advancements in data transmission and storage technologies, such as fifth-generation (5G) networks and big data, these IoT devices have evolved from only performing simple-purpose tasks to supporting multimedia-oriented applications, referred to as Multimedia Internet of Things (M-IoT) [3,4]. M-IoT, especially after being integrated with computer vision and AI, has facilitated significant developments in vision-based monitoring, such as road safety surveillance, smart agriculture, industrial automation, and healthcare services [4]. However, M-IoT is still subject to a variety of constraints that vary across different demands and scenarios. These may involve requirements for high storage or computational capacity, as well as for low power consumption or low latency. Additionally, the increasing resolution of modern devices (e.g., 4K and 8K ultra-high definition (UHD)) has introduced a potential challenge: bandwidth limitations caused by high-bit-rate content transmission [5,6].

To address these challenges, more efficient coding technologies are required [7], highlighting the importance of the High Efficiency Video Coding (HEVC/H.265) standard [8], developed by the Joint Video Experts Team from the ITU-T Video Coding Experts Group and ISO/IEC Moving Picture Experts Group. HEVC has been widely adopted in most smart surveillance devices. However, even HEVC is unlikely to meet the growing demands of next-generation applications. Consequently, its successor, the Versatile Video Coding (VVC/H.266) standard [9], was finalized in July 2020, offering 50% greater coding efficiency than HEVC and providing a solid foundation for high-performance video communication in resource-constrained environments. Several studies [10,11] have validated VVC implementations on hardware platforms (e.g., NVIDIA Jetson) to explore their feasibility in embedded systems.

**Figure 1:**
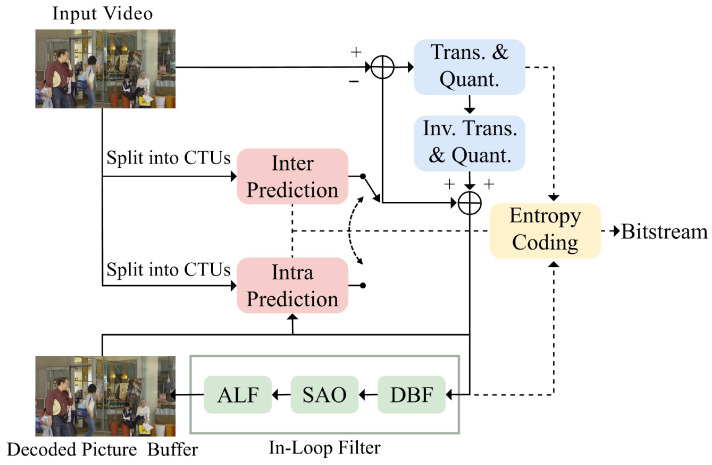
Schematic of in-loop filters in VVC.

Despite the advancements in VVC, block-based hybrid coding methods still face significant challenges. Inevitably, transformation and quantization during the encoding processes result in compression artifacts such as blocking, ringing, and blurring. Blocking artifact removal has seen notable improvements in recent works [12,13,14]. To address these issues, VVC incorporates an in-loop filter, including the deblocking filter (DBF [15]), sample adaptive offset (SAO [16]), and adaptive loop filter (ALF [17]), as shown in Figure 1. This filter sequentially reduces blocking artifacts, mitigates ringing effects, and fine-tunes the image quality. Although these conventional in-loop filters effectively reduce artifacts, they are predefined and cannot fully adapt to the dynamic nature of distortions. As a result, data-driven or AI-enabled approaches have emerged as a promising alternative.

Convolutional neural networks (CNNs) have proven to be powerful tools for numerous low-level vision tasks (e.g., super resolution [18], defogging [19], denoising [20]). Inspired by these successes, researchers have integrated these architectures into the fundamental modules of conventional coding tools, such as intra prediction [21,22,23], inter prediction [24,25,26], CU partitioning [27], and in-loop filtering [28,29,30,31,32,33,34,35,36].

In recent years, CNN-based in-loop filters [31,32,33,34,35,36] have shown significant effectiveness in reducing compression artifacts. However, many of these approaches encounter challenges when adapting to varying QPs. This limitation often requires training separate models for different compression levels, leading to increased time and resource consumption. Thus, several studies [35,36] have been dedicated to addressing this challenge by developing QP-adaptive approaches. However, there remains room for improvement in terms of computational complexity and compression performance.

Transformer-based architectures have gained increased attention in image and video restoration tasks due to their strong ability to model long-range dependencies and capture global contexts. For example, SwinIR [37] demonstrates impressive performance in image restoration by leveraging shifted window-based self-attention. Inspired by such advances, several transformer-based in-loop filtering methods have also been proposed [28,29,30], showing promising improvements in artifact removal and compression quality.

In this paper, we introduce a dual-path residual integrated frequency transformer (DRIFT), an efficient QP-adaptive network incorporating a lightweight frequency fusion convolutional neural network (LFFCNN) as the main processing path and a SwinIR transformer global skip connection (SGS) as the residual path. The experimental results show impressive reductions in the BD rate of 6.56% and 4.83% in the intra and inter modes, respectively. Overall, our primary contributions are as summarized below.

- Our LFFCNN utilizes octave convolution to separate features into high- and low-frequency components, reducing the computational complexity. It incorporates a proposed frequency fusion residual block (FFRB) with four fundamental modules: MSBF for multiscale feature extraction, LFSQAM for QP adaptation, FFM for frequency information exchange, and HAM for the spatial and channel attention mechanism. Notably, LFFCNN achieves comparable BD rate reductions to QA-Filter [36] while reducing the parameter count by 32%.
- LFFCNN enhances local features, whereas SGS leverages Swin transformers to capture long-range dependencies and global contexts. This complementary design significantly improves DRIFT’s performance. Experimental results demonstrate that integrating SGS enhances the BD rate reduction by an additional 1% in intra mode compared to LFFCNN alone.
- A quantization parameter estimator (QPE) is proposed to mitigate double enhancement effects. This addition significantly enhances DRIFT’s coding efficiency, yielding a 0.59% improvement in the BD rate reduction for the inter mode.
- The proposed DRIFT, which is an AI-enabled method, is beneficial in improving the quality of the reconstructed image on M-IOT devices that support VVC standards.

The rest of this paper is organized as follows. Section 2 presents a review of related work, followed by the proposed DRIFT in Section 3. Section 4 shows the experimental results. Finally, Section 5 concludes the article.

## 2. Related Work

### 2.1. CNN-Based In-Loop Filters

CNN-based in-loop filters have made substantial strides in mitigating compression artifacts. As a pioneer, Dong et al. introduced VRCNN [31], employing different kernel sizes within the same layer to extract multiscale features. Kim et al. proposed IACNN [32], expanding the breadth of receptive fields through a parallel network structure. Ding et al. introduced SEFCNN [33] with two serial subnets for feature extraction and enhancement, uniquely addressing the double enhancement issue by selectively applying enhancement at the frame level in low-delay P (LDP) mode and the CU level in random access (RA) mode. Pan et al. proposed EDCNN [34], combining parallel and serial approaches in their feature information fusion block. These diverse architectures demonstrate the potential of CNNs in adaptive filtering for various compression scenarios.

To further improve the generalizability, researchers have explored QP-adaptive methods. X. Song et al. [35] adopted QP maps as additional inputs to better control the filtering strength. However, this approach was limited by its reliance on bias adjustments and input-layer-only QP integration. Liu et al. addressed these limitations with QA-Filter [36], introducing a frequency and spatial QP-adaptive mechanism (FSQAM). This approach integrates QP information into each convolutional layer through weight modulation, allowing for the dynamic adjustment of the filtering strength across the entire network. The FSQAM mechanism represents a significant advancement in QP-adaptive filtering, but there remains room for improvement in the coding efficiency.

### 2.2. Vision Transformer

Vision transformers (ViT) [38] have revolutionized computer vision tasks by leveraging self-attention mechanisms to capture long-range dependencies in images. Whereas traditional CNNs process images through local receptive fields, transformers can model global relationships across the entire image space. However, the computational complexity of a standard ViT scales quadratically with the image size, making it challenging to process high-resolution images effectively. To address this limitation, SwinIR [37] introduces a hierarchical transformer architecture based on shifted windows, which computes self-attention within local windows while maintaining the ability to model cross-window connections through the shifting mechanism.

The effectiveness of SwinIR has been demonstrated across various image restoration tasks [39,40,41]. In this work, we adopt SwinIR with modifications to its mask mechanism to serve as a global skip connection, which we refer to as SwinIR transformer global skip (SGS). The attention mechanism enables the fusion of global context information while preserving local detailed features through the skip connection pathway.

## 3. Proposed Method

**Figure 2:**
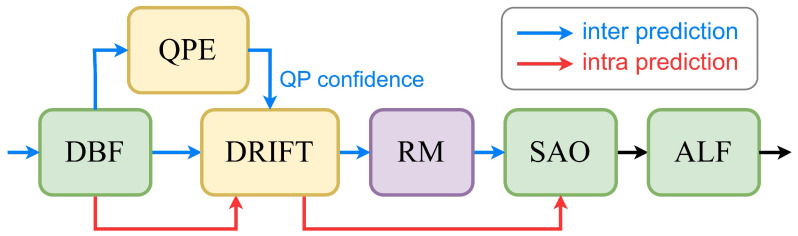
Diagram of in-loop filters integrated with DRIFT in VVC.

Based on the current in-loop filter in VVC, rather than replacing existing components, we integrate our DRIFT between DBF and SAO, as illustrated in Figure 2. Oversmoothing is a common issue for CNN-based filters, especially in inter prediction. To address this, DRIFT leverages QP confidence from our QPE module to refine the input QP values. Additionally, we adopt the residual mapping (RM) module, following the method of Liu et al. [42], to further mitigate double enhancement. This design aims to improve the quality of decoded frames in both intra and inter modes. This section begins with a description of the network architecture, followed by FFRB, and is finalized with details of the proposed QPE.

### 3.1. Network Architecture

**Figure 3:**
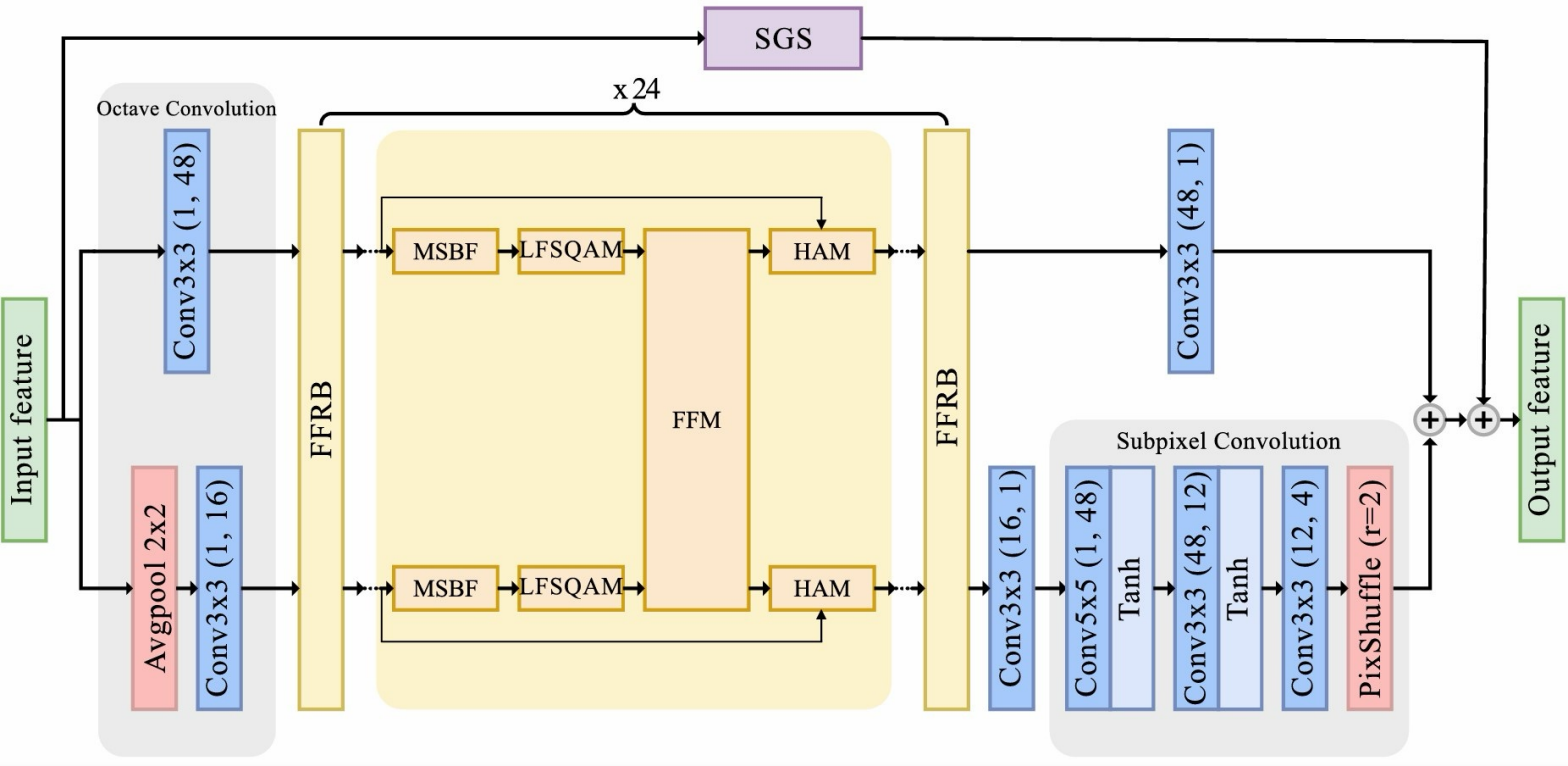
Architecture of DRIFT. DRIFT consists of two components: (1) LFFCNN as the main processing branch, which includes octave convolution, 24 FFRBs, and reconstruction layers, and (2) SGS as the global skip auxiliary path. The final output combines both local and global features.

As shown in Figure 3, the proposed DRIFT consists of two branches: LFFCNN as the main processing branch to enhance local details and SGS as a residual path to capture long-range dependencies. LFFCNN consists of three parts: octave convolution, FFRBs, and reconstruction layers. This architecture mitigates compression artifacts by integrating global and local features.

#### 3.1.1. Octave Convolution

In LFFCNN, the input reconstructed frame is first processed by octave convolution [43] for frequency-based feature decomposition. Let α∈[0, 1] denote the ratio of channels allocated to low-frequency features. The general form of octave convolution processes input features $X_{H}\in R^{\left(1-\alpha_{in}\right)c_{in}\times h\times w},X_{L}\in R^{\alpha_{in}c_{in}\times h\times w}$ into output features $Y_{H}\in R^{\left(1-\alpha_{out}\right)c_{out}\times h\times w},Y_{L}\in R^{\alpha_{out}c_{out}\times h\times w}$ as

(1) $$\begin{array}{cc} Y_{H} & =F_{H\to H}\left(X_{H}^{in}\right)+F_{L\to H}\left(X_{L}^{in}\right)\\ Y_{L} & =F_{H\to L}\left(X_{H}^{in}\right)+F_{L\to L}\left(X_{L}^{in}\right) \end{array}$$

where $F_{H\to H}\left(\cdot \right)$ and $F_{L\to L}\left(\cdot \right)$ represent the intra-frequency convolutions, while $F_{L\to H}\left(\cdot \right)$ and $F_{H\to L}\left(\cdot \right)$ denote the inter-frequency convolutions with upsampling and pooling operations, respectively. Since our model starts with a single input stream, where $X_{L}^{in}=0$, and setting α=0.25, the computation can be simplified to

(2) $$\begin{array}{cc} Y_{H} & =F_{H}\left(x\right)\\ Y_{L} & =F_{L}\left(\mathrm{AvgPool}\left(x\right)\right) \end{array}$$

where $x\in R^{1\times h\times w}$ is the input feature of entire network, while $F_{H}\left(\cdot \right)$ and $F_{L}\left(\cdot \right)$ represent 3 × 3 convolutional layers.

This frequency-based decomposition offers two key advantages compared to direct feature extraction. First, it reduces the computational complexity and memory usage by processing some of the features at a lower resolution. Second, performing convolution on the downsampled features is equivalent to applying a larger receptive field on the original features, enabling the convolutional layers to capture broader spatial contexts without additional computational overhead.

#### 3.1.2. Feature Fusion Residual Blocks

For hierarchical feature learning and the mapping of low-level features to high-level representations, the high- and low-frequency features $Y_{H},Y_{L}$ are parallelly processed through a cascade of 24 FFRBs:

(3) $$\left[Y_{H}^{l+1},Y_{L}^{l+1}\right]=F_{\mathrm{FFRB}}^{l}\left(\left[Y_{H}^{l},Y_{L}^{l}\right]\right)$$

where $Y_{H}^{1}=Y_{H}$, $Y_{L}^{1}=Y_{L}$, and l∈1, 2, …, 24. Let $\left[{\hat{Y}}_{H},{\hat{Y}}_{L}\right]$ denote the final refined features after all FFRBs:

(4) $$\left[{\hat{Y}}_{H},{\hat{Y}}_{L}\right]=\left[Y_{H}^{25},Y_{L}^{25}\right]$$

The detailed architecture of each FFRB will be presented in Section 3.2.

#### 3.1.3. Reconstruction Layers

The refined features are further processed through our reconstruction module, where we employ subpixel convolution for the upsampling of the low-frequency features. For a feature map $X\in R^{c\times h\times w}$, the subpixel convolution operation can be formulated as

(5) $$PS{\left(X\right)}_{c,h,w}=X_{c\cdot r^{2},\left\lfloor h/r\right\rfloor ,\left\lfloor w/r\right\rfloor }$$

where PS(·) denotes the pixel shuffle operation that rearranges the elements of a $H\times W\times C\cdot r^{2}$ tensor to a tensor of shape rH×rW×C, and *r* is the upscaling factor.

Let $f_{k\times k}\left(\cdot \right)$ represent a k×k convolutional layer. The reconstruction process can then be expressed as

(6) $$\begin{array}{cc} \; & {\hat{Y}}_{L}^{\prime }=PS\left(f_{3\times 3}\left({\hat{Y}}_{L}\right)\right)\\ \; & {\hat{Y}}_{H}^{\prime }=f_{3\times 3}\left({\hat{Y}}_{H}\right)\\ \; & Y_{LFFCNN}={\hat{Y}}_{L}^{\prime }+{\hat{Y}}_{H}^{\prime } \end{array}$$

This design leverages subpixel convolution instead of traditional upsampling methods to generate more detailed high-frequency information during the upscaling process. Through channel shuffling into spatial locations, it effectively preserves and enhances fine details in the reconstructed features.

#### 3.1.4. SwinIR Transformer Global Skip

**Figure 4:**
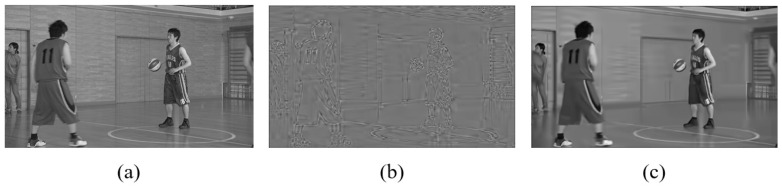
(**a**) Original frame. (**b**) LFFCNN output. (**c**) SGS output.

The SGS branch is adapted from SwinIR [37], with modifications to the window size and embedding dimension to align with the specific requirements of our task. In this work, we adjust them to 8 and 60, respectively. As illustrated in Figure 4c, SGS focuses on capturing long-range dependencies, providing a global structural representation of the entire network input *x*. Its output, $Y_{SGS}$, is combined with the detailed local features $Y_{LFFCNN}$ produced by the LFFCNN branch to form the final output:

(7) $$\begin{array}{cc} \; & Y_{SGS}=F_{\mathrm{SGS}}\left(x_{DRIFT}\right)\\ \; & Y_{\mathrm{DRIFT}}=Y_{SGS}+Y_{LFFCNN} \end{array}$$

This fusion of global and local features leverages the complementary strengths of both branches, resulting in enhanced overall visual quality. Specifically, SGS provides a global coarse structure, while LFFCNN refines local details, addressing compression artifacts effectively.

### 3.2. Details of FFRB

**Figure 5:**
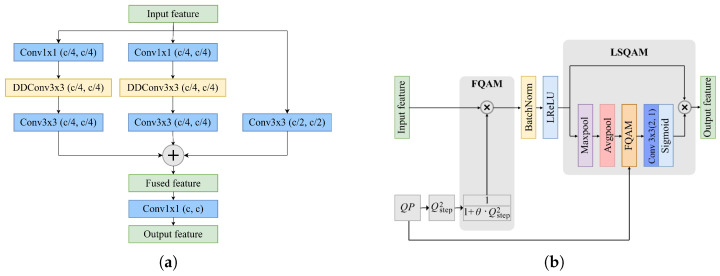
Architectures of (**a**) multiscale branch fusion (MSBF) module and (**b**) lightweight FSQAM (LFSQAM) module.

**Figure 6:**
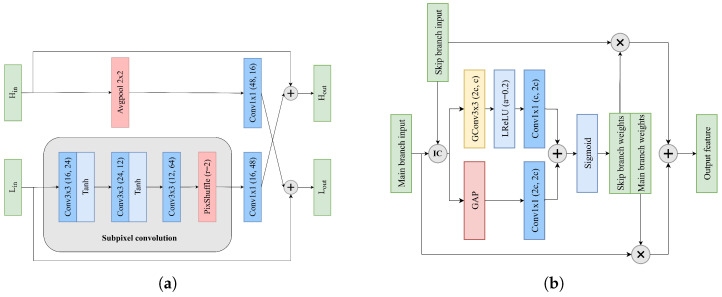
Architectures of (**a**) frequency fusion module (FFM) and (**b**) hybrid attention module (HAM). IC denotes interlaced concatenation.

The FFRB consists of four key modules: multiscale branch fusion (MSBF), the lightweight FSQAM (LFSQAM), a frequency fusion module (FFM), and a hybrid attention module (HAM). The architecture diagrams of each corresponding module are shown in Figure 5 and Figure 6. The following subsections will detail the structure and function of each module.

#### 3.2.1. Multiscale Branch Fusion

Inspired by VRCNN [31], and considering that VVC introduces more diverse block sizes and coding tools than HEVC, which leads to more varied distortion distributions, we propose MSBF. Following the design philosophy of MADNet [44], MSBF extracts features at different scales through three parallel branches. Let $x_{in}\in R^{c\times h\times w}$ denote the input feature; MSBF can be formulated as

(8) $$\begin{array}{cc} \; & F_{left}=f_{3\times 3}\left(f_{d_{2}}\left(f_{1\times 1}\left(x_{in}\right)\right)\right)\\ \; & F_{mid}=f_{3\times 3}(f_{d_{3}}\left(f_{1\times 1}\left(x_{in}\right)\right)\\ \; & F_{right}=f_{3\times 3}\left(x_{in}\right) \end{array}$$

where convolutional layer $f_{1\times 1}\left(\cdot \right)$ reduces the channel dimension from *c* to c/4, and $f_{d_{r}}\left(\cdot \right)$ denotes 3 × 3 depthwise dilated convolution with dilation rate *r*. The output features $F_{left},F_{mid}\in R^{c/4\times h\times w}$ and $F_{right}\in R^{c/2\times h\times w}$ are then fused through

(9) $$y_{fused}=f_{1\times 1}\left(\left[F_{left},F_{mid},F_{right}\right]\right)$$

where [·] denotes channel-wise concatenation. Then, $f_{1\times 1}$ produces the final output feature $y_{fused}\in R^{c\times h\times w}$.

This design brings three main advantages: (1) the parallel branches with different receptive fields effectively capture multiscale feature representations required for handling VVC’s diverse block sizes, (2) the use of dilated convolutions enables larger receptive fields without increasing the computational complexity, and (3) the channel reduction strategy through 1 × 1 convolutions significantly reduces the number of parameters and the computational cost compared to standard convolution, while maintaining feature extraction capabilities.

#### 3.2.2. Lightweight FSQAM

In order to enhance the QP adaptiveness while reducing the parameters, we follow Liu et al.’s [36] FSQAM design and replace the original 5×5 convolution layers with 3×3 ones, which we refer to as LFSQAM. In VVC, the relationship between the QP and the quantization step ($Q_{step}$) is defined as

(10) $$Q_{step}=2^{(QP-4)/6}$$

Here, FSQAM consists of two main components: a frequency QP-adaptive mechanism (FQAM) and a spatial QP-adaptive mechanism (SQAM). Let $z_{in}\in R^{c\times h\times w}$ denote the input feature map. FQAM adaptively adjusts the filtering strength in the frequency domain by decomposing convolution kernels to operate on specific frequencies. The channel-wise scaling factor is introduced as

(11) $$s_{i}=\frac{1}{1+\theta \cdot Q_{step}^{2}},i\in 1,\ldots ,c$$

where θ is a learnable parameter. This scaling factor effectively modulates the convolution operation based on the compression quality:

(12) $$\begin{array}{cc} z_{out} & =\left(w\cdot s\right)*z_{in}+\left(b\cdot s\right)\\ \; & =(w*z_{in}+b)\cdot s \end{array}$$

where *w* and *b* denote the convolution weights and bias terms, respectively. Whereas FQAM considers channel-wise adaptation, SQAM extends the QP-adaptive mechanism to the spatial domain by leveraging maximum and average pooling operations to capture diverse spatial information. Let $z_{sq}$ denote the input feature to SQAM. The spatial attention weights are computed through

(13) $$A=\sigma (f_{5\times 5}\left(\left[MaxPool\left(z_{sq}\right),AvgPool\left(z_{sq}\right)\right]\right))$$

This spatial attention mechanism helps to identify regions requiring different enhancement levels based on local feature characteristics and compression artifacts. The final output feature $\hat{z_{sq}}$ is obtained by

(14) $$\hat{z_{sq}}=A⊙z_{sq}$$

As shown in Figure 5b, our lightweight implementation modifies the original design by replacing the 5 × 5 convolution with a 3 × 3 convolution in LSQAM, reducing the computational complexity while maintaining the adaptive capability for varying compression qualities.

#### 3.2.3. Frequency Fusion Module

Building upon the concept of octave convolution [43], we design an FFM to facilitate the information exchange between high- and low-frequency features obtained from LFSQAM. In contrast to octave convolution, which employs four 3×3 convolutional layers for frequency information interaction, our FFM achieves efficient feature fusion through a more lightweight architecture with only two additional 1 × 1 convolutional layers for channel alignment, alongside the subpixel convolution operations. Let $H_{in}$ and $L_{in}$ denote the input high- and low-frequency features, respectively. The FFM operations can be formulated as

(15) $$\begin{array}{cc} H_{out} & =H_{in}+f_{1\times 1}\left(L_{in}\right)\\ L_{out} & =L_{in}+SubPixelConv\left(H_{in}\right) \end{array}$$

where SubPixelConv(·) denotes the subpixel convolution path consisting of three cascaded convolutional layers followed by a pixel shuffle operation, as illustrated in Figure 6a. This design enables efficient bidirectional communication between frequency components while maintaining relatively low computational complexity.

#### 3.2.4. Hybrid Attention Module

The HAM adaptively adjusts the proportions of original features and learned residual features through a combination of spatial and channel attention mechanisms. Let $x_{skip}$ and $x_{main}$ denote the skip branch input and main branch input, respectively. We first perform an interlaced concatenation operation:

(16) $$X_{ic}=I\left(x_{skip},x_{main}\right)$$

where I(·) represents the interlaced concatenation operation that consists of channel-wise concatenation followed by channel shuffling. The concatenated feature $X_{ic}\in R^{2c\times h\times w}$ is then processed through spatial $F_{s}$ and channel $F_{c}$ attention paths:

(17) $$\begin{array}{cc} F_{s} & =f_{1\times 1}\left(L\left(f_{3\times 3}^{g}\left(X_{ic}\right)\right)\right)\\ F_{c} & =f_{1\times 1}\left(GAP\left(X_{ic}\right)\right) \end{array}$$

where $f_{3\times 3}^{g}$ denotes 3 × 3 group convolution for efficient spatial feature extraction, and L(·) represents the leaky ReLU activation function with negative slope 0.2. Next, $F_{c}$ is upsampled and combined with $F_{s}$ to obtain attention weights:

(18) $$\left[w_{skip},w_{main}\right]=I\left(\sigma \left(F_{s}+UP\left(F_{c}\right)\right)\right)$$

where $w_{skip},w_{main}\in R^{c\times h\times w}$ are the skip branch weights and main branch weights, respectively. Finally, the attention weights are applied to their respective input features to generate the output feature:

(19) $$y_{main}=w_{skip}⊙x_{skip}+w_{main}⊙x_{main}$$

where σ(x) denotes the sigmoid function, ⊙ represents element-wise multiplication, and UP(·) is an unpooling operation.

### 3.3. Quantization Parameter Estimator

**Figure 7:**
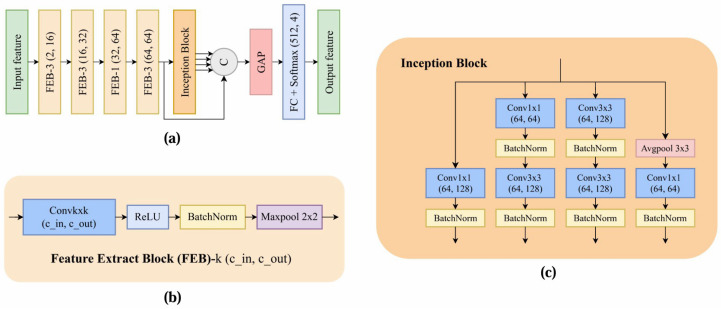
Architecture of QP estimator (QPE). (**a**) QPE. (**b**) FEB. (**c**) Inception block. *C* denotes channel-wise concatenation.

To further mitigate the double enhancement issue in inter mode, we introduce a QPE to calibrate the QP values. Our approach is inspired by Zagoruyko et al.’s [45] two-channel architecture of comparing image patches, which we adapt to estimate the QP values efficiently. As illustrated in Figure 7, the proposed QPE processes deblocked input frames $x_{qpe}$ through four feature extract blocks (FEB), followed by an inception block and fully connected layers for QP confidence estimation. The feature extraction process can be formulated as

(20) $$F_{i}=FEB_{i}\left(F_{i-1}\right),\;i\in 1,\ldots ,4$$

where $F_{0}=x_{qpe}$, and $F_{i}$ represents the intermediate feature maps extracted by the *i*-th FEB with configuration $\left(c_{in},c_{out}\right)$, as shown in Figure 7b. Each FEB contains

(21) $$F_{out}=MaxPool\left(B\left(R\left(f_{k\times k}\left(F_{in}\right)\right)\right)\right)$$

where R(·) represents ReLU activation, and B(·) is the BatchNorm operation. The extracted features $F_{4}$ are further processed through an inception block with multiple parallel convolution paths. Figure 7c details the structure of our inception block. The output features are channel-wise concatenated with $F_{4}$:

(22) $$\hat{F}=\left[f_{1\times 1}\left(F_{4}\right),f_{3\times 3}^{1}\left(F_{4}\right),f_{3\times 3}^{2}\left(F_{4}\right),f_{3\times 3}^{avg}\left(F_{4}\right),F_{4}\right]$$

where $f_{3\times 3}^{1}$ and $f_{3\times 3}^{2}$ represent two different 3 × 3 convolution paths, and $f_{k\times k}^{avg}$ indicates k×k average pooling followed by 1 × 1 convolution. The confidence scores $s_{qp}$ for different QP values are then computed through

(23) $$s_{qp}=\sigma \left(FC\left(GAP\left(\hat{F}\right)\right)\right)\in R^{4}$$

where GAP(·) represents global average pooling, FC(·) denotes fully connected layers, and $s_{qp}$ contains the confidence scores for different QP values. In this work, we estimate 22, 27, 32, and 37. For inter prediction, these confidence scores from all patches are aggregated to determine the overall QP value of the reconstructed frame. This estimated QP is then used to adjust the QP input to DRIFT, mitigating double enhancement effects and potentially improving the overall video coding quality. A comprehensive evaluation of the QPE module’s impact on system performance will be presented in Section 4.5.

## 4. Experimental Results

This section begins by describing the experimental settings in Section 4.1. Section 4.2, Section 4.3, and Section 4.4 present the objective, subjective, and complexity evaluations, respectively. Finally, an ablation study is presented in Section 4.5.

### 4.1. Experimental Settings

For the training process of DRIFT, we utilized the DIV2K dataset [46], which comprises 900 diverse, high-quality RGB images. We allocated 800 images for training and 100 for validation. The dataset was processed using the VVC Test Model (VTM), where the reconstructed images after the deblocking filter served as model inputs, while the original uncompressed images were used as the ground truth. By segmenting the reconstructed images into non-overlapping 64×64 blocks, we obtained 2,250,624 training samples and 285,824 validation samples across four QPs. The model was trained using the mean square error (MSE) as the loss function and implemented using PyTorch 1.12.1, with weights initialized using Kaiming initialization [47] and optimized using the Adam optimizer [48]. Training was completed in approximately 22 epochs with a batch size of 72.

The QPE training utilized 120 videos from the REDS dataset [49], encoded under random access (RA) settings. Patches were labeled based on the mean square error (MSE) between the reconstructed and original frames, where patches with the lowest MSE received a label of 1, and others were assigned 0. The PyTorch model was trained for 100 epochs with a batch size of 64, utilizing the Adam optimizer for all layers.

For evaluation, we integrated the trained models into VTM-12.3 [31] via the LibTorch framework [50]. Following common test conditions (CTC) [51], we tested four QP values (22, 27, 32, and 37) under both all-intra (AI) and random access (RA) configurations. The evaluation used the first 64 frames of the VVC test sequences and was conducted on a system equipped with an Intel i7-10700 CPU and an NVIDIA RTX 3090Ti GPU. To assess the rate distortion performance, the BD rate was calculated based on the PSNR across the four QP settings. This metric reflects the average bit rate savings achieved at equivalent reconstruction quality, serving as a standard indicator for codec efficiency.

### 4.2. Objective Evaluation

To assess the coding performance of our proposed DRIFT, we adopt the BD rate on the luma component as the evaluation criterion. It is found that DRIFT outperforms previous methods [32,33,34,36] in both the AI and RA configurations. Under the AI configuration (Table 1), DRIFT achieved an average BD rate reduction of 6.56% and an up to 10.90% BD rate for sequence BasketballDrill. Under the RA configuration (Table 2), DRIFT attained an average BD rate reduction of 4.83%. Whereas QA-Filter [36] shows a marginal improvement of 0.13% over EDCNN [34] in the RA configuration compared to its more substantial gain in the AI configuration, this performance disparity can be attributed to the impact of double enhancement. In contrast, DRIFT demonstrates consistent improvements of 1.15% and 1.00% over QA-Filter in the AI and RA configurations, respectively, showing promising results in mitigating the oversmoothing issue commonly encountered in in-loop filtering approaches.

### 4.3. Subjective Evaluation

**Figure 8:**
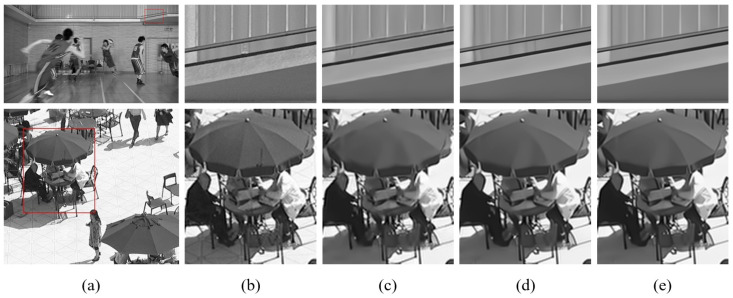
Subjective quality comparison for BasketballDrive (**upper**) and BQSquare (**lower**). (**a**,**b**) Original frame. (**c**) VTM. (**d**) QA-Filter. (**e**) DRIFT.

Subjective quality evaluations were conducted using two video sequences, BQSquare and BasketballDrive, as shown in Figure 8. These sequences were encoded under the random access (RA) configuration with QP=37. We compare the reconstructions from the ground truth (GT), the VTM baseline, QA-Filter [36], and our proposed DRIFT method to assess the visual quality. In the BQSquare sequence, DRIFT demonstrates enhanced structural preservation, particularly in the umbrella frame details, achieving better reconstruction quality compared to QA-Filter. For the BasketballDrive sequence, focusing on wall textures and railing structures, VTM’s reconstruction exhibits noticeable discontinuities in the railing texture, while QA-Filter introduces artifacts in the lower railing region. DRIFT, however, preserves the texture continuity in both the walls and railings without introducing spurious artifacts.

### 4.4. Complexity Evaluation

We analyze the model parameters and encoding time complexity of our proposed methods. Table 3 shows that our LFFCNN achieves a compact model size with 1.22 M parameters, reducing the parameter count by 32% compared to QA-Filter [36]. The complete DRIFT framework, which integrates the LFFCNN and SGS modules, requires 2.09 M parameters. Table 4 compares the encoding time complexity with the VTM benchmark using the GPU acceleration. Our method introduces approximately 8% more encoding complexity compared to QA-Filter. This increase in computational overhead can potentially be attributed to the cascaded residual blocks and data dependencies between modules, as well as the transformer-based computations in the SGS component. Several potential approaches could be explored to address the complexity issue in future work. Model compression techniques such as pruning and quantization could reduce the computational overhead. Inter-layer skipping mechanisms between residual blocks could also be implemented to improve the processing efficiency. These optimizations could potentially maintain the coding performance while reducing the computational demands.

### 4.5. Ablation Study

#### 4.5.1. Effectiveness of QPE

CNN-based loop filters often suffer from double enhancement issues, particularly in inter prediction. To address this, we incorporate the QPE module into our framework. As shown in Table 5, without the QPE, LFFCNN achieves a −4.59% BD rate reduction on average, whereas the complete DRIFT framework further improves this to −5.18%. This improvement demonstrates that our QPE module effectively mitigates the double enhancement problem by providing appropriate QP compensation.

#### 4.5.2. Effectiveness of SGS

Table 6 presents the BD rate analysis results, where LFFCNN without SGS achieves a comparable BD rate reduction (0.23% better) to QA-Filter [36], with fewer parameters. After incorporating the SGS module, our DRIFT framework further improves the coding efficiency to a −6.73% BD rate reduction. This significant improvement of 0.94% in the BD rate reduction validates the effectiveness of our SGS module in capturing global dependencies and enhancing the overall coding performance.

## 5. Conclusions

In this paper, we propose DRIFT, an efficient QP-adaptive in-loop filtering network for VVC, targeting enhanced video quality in bandwidth- and resource-constrained environments such as IoT and AI-enabled embedded systems. First, LFFCNN is introduced, with octave convolution and the FFRB design, achieving a 32% reduction in parameters and with a slight improvement in the BD rate reduction over QA-Filter. Through the integration of a Swin transformer-based global skip connection (SGS), the network’s feature extraction capabilities are further enhanced, contributing an additional 0.92% BD rate reduction in the AI configuration. To address the double enhancement issue in inter prediction, a QP estimator (QPE) is employed to mitigate repeated filtering effects. Overall, DRIFT achieves 6.56% and 4.83% BD rate reductions under the AI and RA configurations, respectively, with an up to 10.90% reduction in the BasketballDrill sequence in intra mode. These results demonstrate that DRIFT offers a powerful and lightweight in-loop filtering solution that enables real-time video processing for AI-enabled systems and M-IOT devices.

In future work, we plan to explore further architectural optimizations to reduce the computational overhead while maintaining the filtering performance. Additionally, broader experimental validation on more diverse datasets and codec configurations will be conducted to assess the generalization capabilities. Finally, potential deployment on hardware platforms (e.g., NVIDIA Jetson) will be investigated to enable real-time operation in embedded and low-power systems. 

## Figures and Tables

**Table 1:** Comparison of BD rate (%) under all-intra (AI) configuration.

Class	Sequence	IACNN [32]	SEFCNN [33]	EDCNN [34]	QA-Filter [36]	DRIFT
A1 (3840 × 2160)	Tango2	−0.97	−2.42	−2.78	−3.82	−5.23
	FoodMarket4	−1.26	−3.82	−3.66	−6.02	−6.93
	Campfire	−0.93	−1.71	−1.92	−2.59	−3.15
A2 (3840 × 2160)	CatRobot1	−1.90	−3.43	−3.77	−5.01	−5.91
	DaylightRoad2	−1.10	−2.08	−2.33	−3.03	−3.97
	ParkRunning3	−1.58	−2.95	−3.26	−4.40	−5.17
B (1920 × 1080)	MarketPlace	−1.60	−2.99	−3.44	−4.50	−5.51
	RitualDance	−3.33	−5.96	−6.50	−8.00	−9.38
	Cactus	−1.70	−2.97	−3.44	−4.47	−5.29
	BasketballDrive	−0.93	−2.38	−2.94	−4.03	−5.53
	BQTerrace	−0.99	−1.76	−2.08	−2.63	−3.54
C (832 × 480)	BasketballDrill	−4.11	−6.86	−7.40	−9.16	−10.90
	BQMall	−3.01	−5.08	−5.59	−6.65	−8.03
	PartyScene	−2.29	−3.38	−3.56	−4.25	−4.98
	RaceHorses	−1.32	−2.10	−2.38	−2.88	−3.95
D (416 × 240)	BasketballPass	−3.80	−6.44	−6.88	−8.19	−9.43
	BQSquare	−3.45	−5.37	−5.66	−6.52	−8.07
	BlowingBubbles	−3.23	−4.73	−4.97	−5.84	−6.78
	RaceHorses	−3.87	−5.15	−5.41	−6.01	−7.11
E (1280 × 720)	FourPeople	−3.39	−5.68	−6.17	−7.55	−9.22
	Johnny	−2.79	−4.87	−5.40	−6.91	−8.31
	KristenAndSara	−2.92	−4.79	−5.26	−6.46	−7.85
Average		−2.29	−3.95	−4.31	−5.41	−6.56

**Table 2:** Comparison of BD rate (%) under random access (RA) configuration.

Class	IACNN [32]	SEFCNN [33]	EDCNN [34]	QA-Filter [36]	DRIFT
A1 Average	−1.52	−3.04	−3.16	−3.07	−3.30
A2 Average	−1.96	−3.17	−3.41	−3.42	−4.56
B Average	−1.64	−2.79	−3.10	−3.44	−3.67
C Average	−1.93	−2.93	−3.13	−3.18	−3.63
D Average	−3.27	−4.63	−4.83	−4.65	−5.45
E Average	−2.70	−4.21	−4.59	−5.22	−5.60
Overall Average	−2.17	−3.46	−3.70	−3.83	−4.83

**Table 3:** Comparison of # parameters.

Method	Parameters (M)
IACNN [32]	0.37
SEFCNN [33]	2.57
EDCNN [34]	18.20
QA-Filter [36]	1.78
LFFCNN	1.22
DRIFT	2.09

**Table 4:** Comparison of encoding time complexity (%).

Class	QA-Filter [36]	DRIFT
B	103.56	110.59
C	103.14	110.00
D	107.45	109.74
E	105.01	118.08
Average	104.79	112.38

**Table 5:** Comparison of BD rate (%) with and without QPE.

Class	LFFCNN	DRIFT
A1	−3.16	−3.79
A2	−5.22	−5.96
B	−3.79	−4.28
C	−3.84	−4.56
D	−5.58	−6.00
E	−5.67	−6.36
Average	−4.59	−5.18

**Table 6:** Comparison of BD rate (%) with and without SGS.

Class	QA-Filter [36]	LFFCNN	DRIFT
A1	−4.31	−4.20	−5.04
A2	−3.03	−3.20	−3.97
B	−4.73	−4.68	−5.85
C	−5.74	−6.10	−6.97
D	−6.64	−7.13	−7.85
E	−6.97	−7.40	−8.46
Average	−5.56	−5.79	−6.73

## Data Availability

The original contributions presented in this study are included in the article.

## References

[1] Kim J., Lee J., Kim J., Yun J. (2013). M2M service platforms: Survey, issues, and enabling technologies. IEEE Commun. Surv. Tutor..

[2] Cao Y., Jiang T., Han Z. (2016). A survey of emerging M2M systems: Context, task, and objective. IEEE Internet Things J..

[3] Floris A., Atzori L. (2016). Managing the quality of experience in the multimedia Internet of Things: A layered-based approach. Sensors.

[4] Nauman A., Qadri Y.A., Amjad M., Zikria Y.B., Afzal M.K., Kim S.W. (2020). Multimedia Internet of Things: A comprehensive survey. IEEE Access.

[5] Bouaafia S., Khemiri R., Messaoud S., Ben Ahmed O., Sayadi F.E. (2022). Deep learning-based video quality enhancement for the new versatile video coding. Neural Comput. Appl..

[6] Choi Y.J., Lee Y.W., Kim J., Jeong S.Y., Choi J.S., Kim B.G. (2023). Attention-based bi-prediction network for versatile video coding (vvc) over 5g network. Sensors.

[7] Guo H., Zhou Y., Guo H., Jiang Z., He T., Wu Y. (2025). A Survey on Recent Advances in Video Coding Technologies and Future Research Directions. IEEE Trans. Broadcast..

[8] Sullivan G.J., Ohm J.R., Han W.J., Wiegand T. (2012). Overview of the high efficiency video coding (HEVC) standard. IEEE Trans. Circuits Syst. Video Technol..

[9] Bross B., Wang Y.K., Ye Y., Liu S., Chen J., Sullivan G.J., Ohm J.R. (2021). Overview of the versatile video coding (VVC) standard and its applications. IEEE Trans. Circuits Syst. Video Technol..

[10] Farhat I., Cabarat P.L., Menard D., Hamidouche W., Déforges O. Energy Efficient VVC Decoding on Mobile Platform. Proceedings of the 2023 IEEE 25th International Workshop on Multimedia Signal Processing (MMSP).

[11] Saha A., Roma N., Chavarrías M., Dias T., Pescador F., Aranda V. (2023). GPU-based parallelisation of a versatile video coding adaptive loop filter in resource-constrained heterogeneous embedded platform. J. Real-Time Image Process..

[12] Lin L., Yu S., Zhou L., Chen W., Zhao T., Wang Z. (2020). PEA265: Perceptual assessment of video compression artifacts. IEEE Trans. Circuits Syst. Video Technol..

[13] Lin L., Wang M., Yang J., Zhang K., Zhao T. (2024). Toward Efficient Video Compression Artifact Detection and Removal: A Benchmark Dataset. IEEE Trans. Multimed..

[14] Jiang N., Chen W., Lin J., Zhao T., Lin C.W. (2023). Video compression artifacts removal with spatial-temporal attention-guided enhancement. IEEE Trans. Multimed..

[15] List P., Joch A., Lainema J., Bjontegaard G., Karczewicz M. (2003). Adaptive deblocking filter. IEEE Trans. Circuits Syst. Video Technol..

[16] Fu C.M., Alshina E., Alshin A., Huang Y.W., Chen C.Y., Tsai C.Y., Hsu C.W., Lei S.M., Park J.H., Han W.J. (2012). Sample adaptive offset in the HEVC standard. IEEE Trans. Circuits Syst. Video Technol..

[17] Tsai C.Y., Chen C.Y., Yamakage T., Chong I.S., Huang Y.W., Fu C.M., Itoh T., Watanabe T., Chujoh T., Karczewicz M. (2013). Adaptive loop filtering for video coding. IEEE J. Sel. Top. Signal Process..

[18] Park S.C., Park M.K., Kang M.G. (2003). Super-resolution image reconstruction: A technical overview. IEEE Signal Process. Mag..

[19] Xu Y., Wen J., Fei L., Zhang Z. (2015). Review of video and image defogging algorithms and related studies on image restoration and enhancement. IEEE Access.

[20] Tian C., Fei L., Zheng W., Xu Y., Zuo W., Lin C.W. (2020). Deep learning on image denoising: An overview. Neural Netw..

[21] Dumas T., Galpin F., Bordes P. (2020). Iterative training of neural networks for intra prediction. IEEE Trans. Image Process..

[22] Park D., Kang D.U., Kim J., Chun S.Y. (2020). Multi-temporal recurrent neural networks for progressive non-uniform single image deblurring with incremental temporal training. European Conference on Computer Vision.

[23] Zhu L., Kwong S., Zhang Y., Wang S., Wang X. (2019). Generative adversarial network-based intra prediction for video coding. IEEE Trans. Multimed..

[24] Huo S., Liu D., Li B., Ma S., Wu F., Gao W. (2020). Deep network-based frame extrapolation with reference frame alignment. IEEE Trans. Circuits Syst. Video Technol..

[25] Murn L., Blasi S., Smeaton A.F., Mrak M. (2021). Improved CNN-based learning of interpolation filters for low-complexity inter prediction in video coding. IEEE Open J. Signal Process..

[26] Pan Z., Zhang P., Peng B., Ling N., Lei J. (2021). A CNN-based fast inter coding method for VVC. IEEE Signal Process. Lett..

[27] Li T., Xu M., Tang R., Chen Y., Xing Q. (2021). DeepQTMT: A deep learning approach for fast QTMT-based CU partition of intra-mode VVC. IEEE Trans. Image Process..

[28] Kathariya B., Li Z., Van der Auwera G. (2023). Joint Pixel and Frequency Feature Learning and Fusion via Channel-wise Transformer for High-Efficiency Learned In-Loop Filter in VVC. IEEE Trans. Circuits Syst. Video Technol..

[29] Kathariya B., Li Z., Wang H., Coban M. Multi-stage spatial and frequency feature fusion using transformer in cnn-based in-loop filter for vvc. Proceedings of the 2022 Picture Coding Symposium (PCS).

[30] Tong O., Chen X., Wang H., Zhu H., Chen Z. Swin Transformer-Based In-Loop Filter for VVC Intra Coding. Proceedings of the 2024 Picture Coding Symposium (PCS).

[31] Dai Y., Liu D., Wu F. (2017). A convolutional neural network approach for post-processing in HEVC intra coding. Proceedings of the MultiMedia Modeling: 23rd International Conference, MMM 2017.

[32] Kim Y., Soh J.W., Park J., Ahn B., Lee H.S., Moon Y.S., Cho N.I. (2019). A pseudo-blind convolutional neural network for the reduction of compression artifacts. IEEE Trans. Circuits Syst. Video Technol..

[33] Ding D., Kong L., Chen G., Liu Z., Fang Y. (2019). A switchable deep learning approach for in-loop filtering in video coding. IEEE Trans. Circuits Syst. Video Technol..

[34] Pan Z., Yi X., Zhang Y., Jeon B., Kwong S. (2020). Efficient in-loop filtering based on enhanced deep convolutional neural networks for HEVC. IEEE Trans. Image Process..

[35] Song X., Yao J., Zhou L., Wang L., Wu X., Xie D., Pu S. A practical convolutional neural network as loop filter for intra frame. Proceedings of the 2018 25th IEEE International Conference on Image Processing (ICIP).

[36] Liu C., Sun H., Katto J., Zeng X., Fan Y. (2022). QA-Filter: A QP-adaptive convolutional neural network filter for video coding. IEEE Trans. Image Process..

[37] Liang J., Cao J., Sun G., Zhang K., Van Gool L., Timofte R. Swinir: Image restoration using swin transformer. Proceedings of the IEEE/CVF International Conference on Computer Vision.

[38] Dosovitskiy A. (2020). An image is worth 16 × 16 words: Transformers for image recognition at scale. arXiv.

[39] Long Y., Wang X., Xu M., Zhang S., Jiang S., Jia S. (2023). Dual self-attention Swin transformer for hyperspectral image super-resolution. IEEE Trans. Geosci. Remote Sens..

[40] Chi K., Yuan Y., Wang Q. (2023). Trinity-Net: Gradient-guided Swin transformer-based remote sensing image dehazing and beyond. IEEE Trans. Geosci. Remote Sens..

[41] Fan C., Liu T., Liu K. (2022). SUNet: Swin transformer UNet for image denoising. arXiv.

[42] Liu C., Sun H., Katto J., Zeng X., Fan Y. (2020). A convolutional neural network-based low complexity filter. arXiv.

[43] Chen Y., Fan H., Xu B., Yan Z., Kalantidis Y., Rohrbach M., Yan S., Feng J. Drop an octave: Reducing spatial redundancy in convolutional neural networks with octave convolution. Proceedings of the IEEE/CVF International Conference on Computer Vision.

[44] Lan R., Sun L., Liu Z., Lu H., Pang C., Luo X. (2020). MADNet: A fast and lightweight network for single-image super resolution. IEEE Trans. Cybern..

[45] Zagoruyko S., Komodakis N. Learning to compare image patches via convolutional neural networks. Proceedings of the IEEE Conference on Computer Vision and Pattern Recognition.

[46] Agustsson E., Timofte R. Ntire 2017 challenge on single image super-resolution: Dataset and study. Proceedings of the IEEE Conference on Computer Vision and Pattern Recognition Workshops.

[47] He K., Zhang X., Ren S., Sun J. Delving deep into rectifiers: Surpassing human-level performance on imagenet classification. Proceedings of the IEEE International Conference on Computer Vision.

[48] Kingma D.P. (2014). Adam: A method for stochastic optimization. arXiv.

[49] Nah S., Baik S., Hong S., Moon G., Son S., Timofte R., Lee K.M. NTIRE 2019 Challenge on Video Deblurring and Super-Resolution: Dataset and Study. Proceedings of the The IEEE Conference on Computer Vision and Pattern Recognition (CVPR) Workshops.

[50] Paszke A., Gross S., Massa F., Lerer A., Bradbury J., Chanan G., Killeen T., Lin Z., Gimelshein N., Antiga L. (2019). Pytorch: An imperative style, high-performance deep learning library. Adv. Neural Inf. Process. Syst..

[51] Suehring K., Li X. (2017). Common Test Conditions and Software Reference Configurations, document JVET-G1010.

